# Pharmacologic management of weight regain following bariatric surgery

**DOI:** 10.3389/fendo.2022.1043595

**Published:** 2023-01-09

**Authors:** Eugene Lucas, Okeefe Simmons, Beverly Tchang, Louis Aronne

**Affiliations:** Weill Cornell Medical Center, New York-Presbyterian, New York, NY, United States

**Keywords:** bariatric (weight loss) surgery, anti-obesity medications, obesity, weight regain after bariatric surgery, obesity pharmacotherapy

## Abstract

While bariatric surgery restults in significant long-term weight loss for most patients with obesity, post-surgical weight gain affects a considerable percentage of patients to varying degrees of severity. Furthermore, a small but significant percentage of patients experience inadequate post-surgical weight loss. Although many studies have examined the role of anti-obesity medications to address post-operative weight regain, an evidence-based consensus has not yet been achieved because of the heterogeneity of populations studied and the studies themselves. Observational studies in the post-bariatric surgery population consistently demonstrate the benefit of medical weight management after bariatric surgery, with most evidence highlighting liraglutide, topiramate, and phentermine/topiramate. New anti-obesity medications are anticipated to be helpful for post-surgical weight optimization given their efficacy in the non-surgical population.

## Introduction

Bariatric surgery has been demonstrated to achieve significant weight loss in patients with obesity. The sleeve gastrectomy (SG) and Roux-en-Y gastric bypass (RYGB) have historically been the most popular bariatric surgical procedures in the US and remained so in 2019 at 61.4% and 17.0% respectively, with bariatric revision of a prior procedure ranking 3rd most common at 16.7% (1). Multiple definitions of what constitutes significant post-bariatric weight regain and inadequate post-bariatric procedure weight loss have been used in the literature, which can make comparisons difficult. Nevertheless, the heterogeneous studies can inform an evidence-based approach for the evaluated groups of patients.

Various studies have reported on the magnitude, timing, and durability of weight loss following bariatric surgeries. The ongoing Longitudinal Assessment of Bariatric Surgery (LABS) prospective observational study examined 1406 individuals after RYGB who were followed up for 5 years or longer and found that weight regain (WR) occurred as early as within one year of RYGB after the nadir weight was achieved, with patients regaining a median of 26.8% of maximum weight loss at 5 years after surgery, or a median weight regain of 9.7% relative to their presurgical weight at 5 years after surgery (2). In the landmark prospective observational Swedish Obese Subjects (SOS) study, 2010 individuals who had undergone bariatric surgery were compared to contemporaneously matched control subjects with obesity (3, 4). The average maximal weight loss following gastric banding, vertical banded gastroplasty (VGB), and RYGB was 21-38% and occurred around 1-2 years post-procedure. Patients experienced gradual weight regain totaling 8-13% before reaching a weight loss plateau (WLP) at 8-10 years after the procedure. The largest observational study of bariatric surgery patients was conducted by Baig et al. who reviewed 9617 patient charts from 26 weight centers in India to characterize clinically significant post-bariatric WR at 5 years following SG, RYGB, and one-anastomosis gastric bypass (OAGB). Mean WR ranged from 6-22% at 5 years depending on type of bariatric surgery (5). The percentage of patients who experienced WR at 5 years following bariatric surgery ranged from 15.9% to 35.1% for SG, 5% to 14.6% for RYGB, and 1% to 3% for OAGB, with percentages varying depending on the definition used for WR in the study.

The LABS, SOS and Baig et al. studies highlight that WR following bariatric surgery is not uncommon and affects patients to varying degrees of severity depending on the type of bariatric surgery performed. WR can be a distressing experience for patients, and excess weight regain is associated with many diseases, such as diabetes, hypertension, hyperlipidemia, asthma, arthritis, depression, coronary heart disease, and various malignancies (6). Therefore, it is incumbent upon weight management providers to offer interventions to prevent post-bariatric surgery weight regain and optimize the weight status of such patients. Anti-obesity medications (AOMs) have been investigated in a variety of contexts related to type of bariatric surgery, the specific AOM or combination of AOM used, and the timing of AOM initiation and are evidence-based options for post-bariatric surgery weight optimization.

## Pharmacologic management for post-bariatric surgery weight optimization

Medical management of obesity is recommended for individuals with a body mass index (BMI) of 30 kg/m^2^ or greater and for individuals with a BMI of 27 kg/m^2^ plus comorbidities (7). Pharmacologic agents can facilitate weight loss by opposing various physiologic mechanisms that contribute to obesity (8). Currently, five medications are FDA-approved for the long-term treatment of obesity: orlistat, phentermine/topiramate, liraglutide 3.0mg, naltrexone/bupropion, and semaglutide 2.4mg. Several medications are used off-label for long-term weight management: phentermine (>3 months), topiramate, liraglutide 1.8mg, naltrexone, bupropion, semaglutide 2.0mg, and tirzepatide. Their effectiveness in treating post-bariatric weight regain remains an area of active investigation.

**Table 1** summarizes studies that evaluated the weight loss effect of AOMs in patients who underwent bariatric surgery. All results in **Table 1** achieved statistical significance within referenced study unless otherwise specified.

**Table 1:** Summary of Studies on Pharmacotherapies Aimed at Addressing Weight Gain After Bariatric Surgery.

Study	Methods	Study Characteristics	Weight Outcomes			Comorbidity Analysis	Other Details
Liraglutide Studies							
Pajecki 2013 {23912365}	Retrospective study of 15 patients with WR or EWL of <50% after 2 years post-op investigated WL associated with Lira WR defined as > 15% of nadir	Surgery Types RYGB (n=9) GB (n=4) DSBPD (n=1) SG (n=1) Study Dur. 4.2 months BP to AOM 5.3 ± 3.3 years	Weight Change			Not applicable	53% of patients received Lira 1.8mg/day. Lira was used for mean duration of 12.5 ± 4.7 (range: 8-28) weeks.
			-7.3%				
			EWL				
			66.7 ± 22.4% (41.4 - 112.9%)				
Gorgojo-Martinez 2016 {27256860}	Retrospective study of 15 patients with WR investigated WL associated with Lira compared to 149 patients with no prior bariatric surgery WR defined as > 5% from nadir	Surgery Types RYGB (n=8) BPD (n=3) VBG or LAGB (n=4) SG (n=1) Study Dur: 24 months BP to AOM 5.2 years	Weight Change			Patients with a history of bariatric surgery had a smaller reduction in A1c in response to Lira than those without bariatric surgery (-0.39% vs. -0.67%)	Significantly more non-surgical patients discontinued liraglutide due to all causes (34.9% versus 6.7%).
			Post-bariatric surgery -3.4kg	No bariatric surgery -3.8kg			
Miras 2019 {31174993}	RCT of Lira vs. placebo in 80 patients with persistent or recurrent DM2 who had undergone RYGB or VSG	Surgery Types RYGB (n=61) VSG (n=19) Study Dur.: 26 weeks BP to AOM: 38 months	Weight Change Lira vs. Placebo			Lira use was associated with an A1c change of -1.22% relative to placebo No statistically significant difference between the lira and placebo groups in systolic and diastolic blood pressure, or lipids at week 26	Type of bariatric surgery had no significant effect on the outcome. Maximum dose of Lira used was 1.8mg/day. Weight change was analyzed as a secondary endpoint.
			-4.23 kg				
Suliman 2019 {30768836}	Prospective study of 2092 patients compared WL associated with Lira 3mg/d in patients with and without a history of a bariatric procedure	Surgery Types RYGB (n=47) SG (n=118) Other (n=23) Study Dur. 28 weeks BP to AOM 4 years	Weight Change at ≥; 16 weeks			Not applicable	Study population: 93% Emirati Arabs, 7% other Arabs Of 2092 patients treated with lira 3 mg, 425 patients (20%) stopped the drug after a median period of 108 days, with side effects specified as the reason for stopping the drug in 140 patients (6.7% of the total cohort).
			Post-bariatric surgery	No bariatric surgery			
			-6.4%	-6.1%			
			Weight Change at 28 weeks in post-surgical population (n=340)				
			Overall	RYGB	SG		
			-7.6%	-5.6%	-3.3%		
Wharton 2019 {31183988}	Retrospective study of 117 patients with WR investigated WL associated with Lira among different bariatric surgeries	Surgery Types RYGB (n=53) SG (n=14) GB (n=40) Study Dur. 38.7 months BP to AOM 7.8 ± 5.7 years	Weight Change			Not applicable	Patients experienced a significant WL 1-2 months after initiation of Lira 3.0 mg. WL remained significant up to 1 year of use with Lira, regardless of the type of bariatric surgery. Within-group weight changes were significant compared to baseline. Between-group weight changes were not statistically significant.
			RYBG	LSG	GB		
			-6.6% ± 7.1%	-4.9% ± 5.6%	-3.6% ± 3.0%		
Horber 2020 {32691401}	Prospective study of 65 patients with WR compared WL associated with Lira 3.0mg, endosurgery (ES), or Fobi-Ring (FR) WR defined as > 10% from nadir	Surgery Type RYGB (n=65) Study Dur. 24 months BP to AOM >6 years	Weight Change			Prevalence of hypertension and dyslipidemia decreased in the Lira and FR groups at 24 months	Lira median daily tolerated dose of 2.0 mg, range 0.6 - 3.0 mg Patients in the Lira group lost 87% of weight gained from weight nadir at 10-11 years after RYGB. Results for weight change achieved statistical significance when compared with patient’s weight before beginning intervention.
			Lira	ES	FR		
			-13 ± 8 kg	-3 ± 3 kg	-17 ± 7 kg		
Thakur 2021 {32656729}	RCT of 30 patients with BMI > 30 who underwent LSG evaluated WL in response to post-op Lira vs. placebo	Surgery Type SG (n=30) Study Dur 6 months BP to AOM 6 weeks	Weight Change			There were significant improvements in glycemic, blood pressure, and lipid parameters at 24 weeks compared to baseline with no intergroup differences No significant difference in resolution of other obesity-related comorbidities between two groups at 24-weeks	Intergroup weight changes were not statistically significant (p=0.116). EWL with Lira was significantly greater than Placebo (p=0.043)
			Lira	Placebo			
			-28.2 ± 5.7%	-23.2 ± 6.2%			
			EWL				
			Lira 58.7 ± 14.3%	Placebo 44.5 ± 8.6			
Phentermine, Topiramate, and Phentermine + Topiramate Studies							
Zilber-stein 2004 {15318986}	Prospective study of 16 patients with inadequate weight loss or WLP investigated WL associated with Topiramate	Surgery Type AGB (n=16) Study Dur. 90 days BP to AOM 5-13 months	Mean Weight Change -7.1 kg			Not applicable	Topiramate doses: 12.5mg (n=2), 25mg (n=10), 50mg (n=2) 10 of 16 patients reported binge eating episodes prior to surgery. Weight change was not assessed for statistical significance.
			EWL 13.2% (range -3% to -19%)				
Schwartz 2016 {26615406}	Retrospective study of 65 patients with WR or WLP investigated WL associated with Ph or Ph-T	Surgery Types RYGB (n=51) LAGB (n=14) Study Dur 90 days BP to AOM 36 months	Weight Change			Those whose DM2 and hypertension had not resolved by the start of pharmacotherapy did not see resolution of DM2 and/or hypertension at 90 days post-pharmacotherapy	Statistical analysis of intergroup weight changes was not reported. After adjustments, patients on Ph weighed 1.35 kg less than those on Ph-Top. No significant WL differences were observed between RYGB or LAGB.
			Ph 37.5 mg (n=24)	Ph-Top 7.5-46 mg (n=6)			
			-6.35 kg, 12.8% EWL	-3.81 kg, 12.9% EWL			
Ard 2019 {31147285}	Open-label single-arm trial in 13 patients with BMI > 50 who were started on Ph-Top at 3 months pre-op and continued for 2 years post-op investigated WL associated with Ph-Top compared to 40 matched historical controls who did not receive Ph-Top	Surgery Type SG (n=13) Study Dur: 27 months BP to AOM 3 months pre-BP	Weight Change			Not applicable	Least square mean difference in weight change between groups was significant at -11.16% (p=0.007). All patients were initiated on Ph-Top at 3.75/23 mg capsule once daily for 2 weeks, then increased to 7.5/46 mg daily. Patients changed regimen to Ph-Top to every other day dosing 2 weeks before BP and stopped 7 days before Ph-Top. Patients resumed Ph-Top at 7.5/46 mg daily approximately 1 month after BP.
			Ph-Top	No Ph-Top			
			-38.2 kg (-45.4 to -31.0)	-27.00 kg (-31.0 to -23.0)			
Orlistat Study							
Zoss 2002 {11868286}	Open-label uncontrolled non-randomized trial of 38 patients with WLP evaluated WL in response Orlistat 120mg TID + low-fat diet vs. low-fat diet alone	Surgery Type AGB (n=38) Study Dur. 8 months BP to AOM 18 ± 6 months	Weight Change			Not applicable	15 patients were enrolled in a 9-month extension study, of which 8 remained on orlistat and.7 discontinued; weight remained unchanged regardless of orlistat exposure.
			Orlistat + diet -8 ± 3 kg	Diet alone -3 ± 2 kg			
Multiple AOM Comparison Studies							
Hanipah 2017 {29287757}	Retrospective study of 209 patients with WR or inadequate WL investigated WL associated with AOMs AOMs: Ph (n=156), Ph-Top (n=25), Lor (n=18), Nal-Bup (n=10)	Surgery Types RYGB (n = 126) SG (n = 52), LAGB (n = 21), Other (n = 10) Study Dur. 12 months BP to AOM: 38 months	Weight Change			Not applicable	Results for weight change achieved statistical significance for LAGB vs SG and for RYGB vs SG. 12 of 45 patients with DM2 were concurrently on Lira 1.2-1.8 mg/d. Weight changes according to individual AOM exposure was not reported. 37% of patients on at least 1 AOM achieved additional WL of >5%, and 19% of patients achieved an additional WL of >10% at 1 year (statistical significance not reported). Weight change for patients with BMI > 36 was 3.5 ± 7.9% and for patients with BMI < 36 was 0.9 ± 7.0%.
			RYGB	SG	GB		
			-3.2 kg -4.6% WL	-0.3 kg -0.3% WL	-4.6 kg -4.6% WL		
Stanford 2017 {27986587}	Retrospective study of 319 patients with WR or inadequate weight loss and exposed to 1 or more AOMs AOMs evaluated: Ph, Top, Ph-Top, Metf, Bup, Bup-Nal, Orlistat, Sibutramine, Lira, Exenatide, Pramlintide, Nal, Lor, Canagliflozin, Zonisamide	Surgery Types RYGB (n=258) SG (n= 61) Study Dur. 12 months BP to AOM RYGB: 59.3 months; SD = 36.7 SG: 23.2 months; SD = 15.3	Weight Change			Patients who had one obesity co-morbidity at the time of their bariatric procedure were less likely to lose ≥ 15% of their preoperative weight with AOMs after their bariatric procedure. OSA and psychiatric comorbidities were associated with less weight loss. DM2 was not a predictor of WL.	Pts prescribed AOMs at their WLP achieved 32.3% WL vs 26.8% when AOMs were prescribed after experiencing WR. Patients taking Top were twice as likely to lose > 10% of their weight relative to other AOMs. Patients who had RYGB were more likely to lose > 10% of their weight with AOMs. WL results with Ph, Metf, Bup, Zonisamide were not statistically significant.
			Top	AOMs other than Top			
			-20.2 ± 24.5 lbs	-13.99 ± 13.6 lbs			
Stanford 2018 {30595995}	Retrospective study of 35 patients aged > 60 years with WR or inadequate weight loss examined WL associated with AOMs AOMs evaluated: Same as Stanford 2017	Surgery Types RYGB (n=24) SG (n=11) Study Dur. 12 months BP to AOM 34.6 months (SD=25.3)	BMI Change			Pre-operative hypertension was a negative predictor of AOM-associated WL.	No statistically significant difference was detected in BMI change when AOMs were prescribed at time of WLP (n=10) vs. at the time of WR (n=25). Liraglutide was the only AOM that predicted WL. Weight changes according to individual AOM exposure was not reported.
			RYGB	SG			
			-3.37 ± 2.83	-1.38 ± 1.49			
Toth 2018 {30158481}	Retrospective study of 37 post-bariatric surgery patients aged 21-30 years examined WL associated with type and timing of AOM usage AOMs evaluated: Ph, Top, Metf WLP was defined as weight within 3% of nadir WR was defined as > 3% increase from nadir	Surgery Types RYGB (n=28) SG (n=9) Study Dur. 14 years BP to AOM Overall: 52.2 months (SD = 38.7) LSG: 20.1 ± 5.2 months RYGB 62.6 SD 39.1)	Weight Change			Not applicable	RYGB was associated with more WL on AOMs (−8.1%) compared to SG (−3.3%) with the differences approaching statistical significance. No statistically significant difference found for AOM regimens with Ph vs those w/o Ph and those w/ Top vs those w/o Top.
			AOM Initiated at WLP	AOM Initiated at WR			
			-41.2%	-27.1%			
			AOM regimen w/ Metformin	AOM regimen w/o Metformin			
			-2.9%	-7.7%			
Srivastava 2018 {29464536}	Retrospective study of 48 patients with WR examined WL among patients exposed to AOM and behavior therapy compared to 48 matched controls with no bariatric procedure history AOMs: Ph, Top, Ph-Top, Metf, Bup, Bup-Nal, Lor, Zoni, GLP-1-RA	Surgery Types RYGB (n=25) SG (n=14) LAGB (n=9) Study Dur. 20 years BP to AOM 6.1 years ± 4.6 years	Time/ Intervention	Weight Change		Not applicable	No statistical significance was identified in assessing AOM associated WL according to bariatric procedure type. No statistical significance was identified at 6 months in the Non-PBP group taking 1 AOM vs the Non-PBP group on no AOM. A non-statistically significant trend toward greater WL was identified in the PBP group taking ≥ 2 AOMs relative to the PBP group on 1 AOM or no AOM.
				PBP	Non-PBP		
			3 months	-2.2%	-4.0%		
			6 months	-4.2%	-7.3%		
			>2 AOMs	-5.7%	-9.5%		
			1 AOM	-2.7%	-6.8%		
			No AOM	-2.2%	-4.8%		
Istfan 2020 {32441476}	Retrospective study of 760 patients with WR examined WL associated with adherence to AOMs vs. no AOMs AOMs: Ph, Top, Lor, Ph-Top, Nal-Bup	Surgery Type RYGB (n=760) Study Dur. 11 years BP to AOM 6 years	Hazard Ratio for Pts with WR of 1.22%/month			No statistically significant difference in the prevalence of comorbidities was identified among AOM users versus non-AOM users.	Results for the AOM Non-Adherent patients did not reach statistical significance. AOM Adherent: Patients prescribed an AOM and who arrived for an office visit within 60 days of the date of the AOM prescription. AOM Non-Adherent: Patients prescribed an AOM and who did not return for an office visit within 60 days. No AOM: Patients without an AOM prescription.
			AOM Adherent	AOM Non-Adherent	Non AOM Users		
			0.729	0.844	1		
Edgerton 2021 {33420652}	Retrospective study of 197 patients with WR, adequate WL, or inadequate WL, evaluated WL associated with AOMs AOMs: Ph, Top, Ph-Top, Bup, Bup+Nal, Metf, Top-Metf, Lira, Dulaglutide	Surgery Types RYGB (n=72) SG (n=125) Study Dur. 3 years BP to AOM 65.6 ± 106.5 months	Weight Change			A greater percentage of patients had HTN in the WR group (41.5%) versus the inadequate WL group (27.8%). A smaller percentage of patients had DM2 in the WR group (19%) than in the inadequate WL group (38.9%).	Weight change reported at mean 11.2 (± 8.4) months. WL on AOMs was significantly higher for patients with RYGB than SG. Mean WL was significantly greater for Ph-Top compared with Ph and for GLP-1-RA compared with Ph; other comparisons did not reach statistical significance. 48% patients were classified as having changed AOM regimen whereas 52% did not.
			Ph-Top	Ph			
			-9.8% ± 5.6	-4.5% ± 3.7			
			GLP-1-RA	Other AOMs			
			-7.7% ± 6.0	-6.2% ± 4.8			
Gazda, 2021 {33818009}	Retrospective study of 207 patients with a history of a bariatric surgery assessed WL associated with AOMs AOMs: Orlistat, Top, Ph, Ph-Top, Lisdexamphetamine, Bup, Bup-Nal, Lorcaserin, Sema, Exenatide, Dulaglutide, Albiglutide, Lixisenatide	Surgery Types SG/Gastroplasty (n = 80) RYGB/ BPDDS (n = 73) GB (n = 54) Study Dur. 12 months BP to AOM 7.3 ± 5.9 years	Weight change according to Intervention at 9 months			The presence of diabetes at the time of the patient enrollment in either ILM or AOM-based therapy was not found to be a significant predictor of weight change.	GLP-1 Receptor Agonists (GLP-1-RA) were superior to non-GLP-1-RA based therapies and to ILM at 3 months, 6 months, and 9 months following the initial visit to address WR. Authors did not include more granular data on the weight loss reductions achieved on specific GLP-1-RA agents or the dosages utilized. Within the GLP-1-RA group, the authors noted that only 13% of patients were prescribed a non GLP-1-RA medication for the purpose of weight loss.
			ILM	Non–GLP-1	GLP-1		
			-1.6% ± 12.6%	-5.6% ± 9.9%	-6.9% ± 6.9%		

AGB, Adjustable Gastric Banding (AGB); Bup, Bupropion; Bup-Nal, Bupropion- Naltrexone; DSBPD, Duodenal Switch Biliopancreatic Diversion; Lira, Liraglutide; Metf, Metformin; PBP, Post-Bariatric Procedure; WLP, Weight Loss Plateau; Ph, Phentermine; Ph-Top, Phentermine-Topiramate; Pram, Pramlintide; Sema, Semaglutide; Top, Topiramate; WR, Weight Regain.

### Orlistat

Orlistat is an intestinal lipase inhibitor that reduces fat absorption by approximately 30% (9) FDA-approved in 1999 for the treatment of obesity, it is available as an over-the-counter medication at doses up to 60 mg three times per day and as a prescription at doses up to 120 mg three times per day. Orlistat was associated with weight loss in a non-randomized intervention study of 38 patients experiencing a weight loss plateau (WLP) after adjustable gastric banding, with a weight change of -8 ± 3 kg at 8 months compared to -3 ± 2 kg with dietary intervention alone (10). Although not assessed for statistical significance, patients on orlistat in this study reported improvement of constipation symptoms.

### Phentermine, topiramate, and phentermine/topiramate

Phentermine is a sympathomimetic that suppresses appetite *via* central neural pathways (11). Topiramate is an anti-epileptic that also has central anorexigenic effects with benefits demonstrated in binge eating disorder (12, 13). The combination phentermine/topiramate was FDA-approved for the treatment of obesity in 2012. Phentermine, topiramate, and phentermine/topiramate benefit from a large body of evidence supporting their use in patients with a history of bariatric surgery (14–18). In the largest retrospective study of 319 patients, topiramate was associated with the greatest weight change (-20.2lbs) after RYGB or SG as compared to 15 other AOMs (-13.99 lbs. for AOMs other than topiramate) (17). However, this signal for topiramate was not observed in a subgroup of patients ages 21-30 years (19). A similar study of 197 post-surgical patients reported that phentermine/topiramate was associated with the greatest odds of achieving 5, 10, and 15% weight loss compared to other AOMs (20). Adherence to topiramate, phentermine, or combination phentermine/topiramate has been consistently associated with greater weight loss (18). Among 16 patients with binge eating disorder and a history of bariatric surgery, topiramate 12.5-50mg per day was associated with an additional excess weight loss of 13.7% (14). In a retrospective study of 30 patients, when phentermine 37.5mg was compared with phentermine-topiramate 7.5-46mg, both produced significant weight losses of 6.3 kg and 3.8 kg, respectively, over 90 days, and phentermine 37.5mg was statistically superior (15). Finally, in patients with a BMI > 50 who underwent laparoscopic sleeve gastrectomy, initiation of phentermine-topiramate 7.5-46 mg at 3 months pre-operatively was found to facilitate superior weight loss results relative to historically matched controls who did not receive phentermine-topiramate 7.5-46 mg (16).

### Liraglutide

Liraglutide is a glucagon-like-peptide-1 receptor agonist (GLP-1-RA) and is FDA-approved for type 2 diabetes as liraglutide 1.8mg and for obesity as liraglutide 3.0mg. Benefit of liraglutide in the post-bariatric surgery setting is supported by a randomized controlled trial (21) and multiple observational studies (22–27). The randomized double-blind placebo-controlled trial, which was performed to investigate the effect of liraglutide 1.8mg for the management of patients with a history of bariatric surgery and persistent or recurrent type 2 diabetes, reported as a secondary endpoint a statistically significant mean weight change of -4.23 kg with liraglutide vs. placebo at 26 weeks (21). Of the observational studies, the largest included 787 patients treated with liraglutide 3.0 mg for ≥ 16 weeks and demonstrated a weight change of -6.4% for patients with a history of bariatric surgery versus -6.1% for patients without a history of bariatric surgery (24). Use of liraglutide in patients with a history of bariatric surgery has also been associated with improvements in blood pressure (25) and hemoglobin A1c (21, 26). In comparing the effect of liraglutide 3.0 mg in patients with different bariatric surgeries, those who had a history of RYGB lost significantly more weight (-5.6%) than patients who had a history of SG (-3.3%) (24); similar results have been replicated (-6.6% with RYGB vs. -3.6% with SG) but were not found to be statistically significant (25). Furthermore, there were significantly fewer discontinuations of liraglutide in patients with a history of bariatric surgery compared to those without a history of bariatric surgery (26).

### Naltrexone, bupropion, and naltrexone/bupropion

Naltrexone is an opioid receptor antagonist that inhibits the auto-inhibition of anorexigenic neurons in the hypothalamus (28). Bupropion is a dopamine and norepinephrine reuptake inhibitor that can suppress appetite (28). The combination naltrexone/bupropion was FDA-approved for the treatment of obesity in 2014. There are no studies evaluating the individual efficacies of naltrexone, bupropion, or naltrexone/bupropion specifically in the post-bariatric surgery population. A few studies have investigated the effectiveness of obesity pharmacotherapy in general, in which monotherapies naltrexone or bupropion and combination naltrexone/bupropion were minimally represented (17, 29, 30). The strongest body of evidence for weight loss efficacy stems from phase 3 randomized controlled trials of combination naltrexone/bupropion, from which individuals with a history of bariatric surgery were excluded, but demonstrated about 5% placebo-subtracted weight loss at one year (31, 32).

### Semaglutide

Semaglutide is a GLP-1-RA that is FDA-approved for type 2 diabetes as semaglutide 2.0mg and for obesity as semaglutide 2.4mg. There are no studies evaluating semaglutide specifically in the post-bariatric surgery population. Several phase 3 randomized controlled trials, which excluded individuals with a history of bariatric surgery, proved superior weight loss with semaglutide 2.4mg compared to placebo in individuals with obesity or metabolically complicated overweight: 12.4% in general (33), 11.1% in East Asian populations (34), 6.2% in individuals with type 2 diabetes (35), and 13.3% in the setting of intensive lifestyle modification (36). A double-blinded, randomized, placebo-controlled trial of semaglutide 2.4mg in patients with inadequate weight loss following bariatric surgery is underway (BARI-STEP) (37).

### Tirzepatide

Tirzepatide is a dual GLP-1-RA and gastric inhibitory peptide receptor agonist (GIP-RA). It is FDA-approved for the treatment of type 2 diabetes. There are no studies evaluating tirzepatide specifically in the post-bariatric surgery population. However, a recent phase 3 trial, which excluded individuals with a history of bariatric surgery, demonstrated weight loss efficacy approaching that of some bariatric surgeries. In a double-blind, randomized, controlled trial of 2539 adults with obesity (BMI ≥ 30) or medically complicated overweight (BMI ≥ 27), tirzepatide 15mg weekly resulted in 20.9% weight loss over 72 weeks compared to 3.1% weight loss with placebo (38). More than half of participants on tirzepatide 15 mg achieved weight loss of ≥ 20%: 57% vs. 3% with placebo. Reduction in body weight of ≥ 25% was observed in 36% of participants on tirzepatide 15 mg vs. 1.5% on placebo.

### General obesity pharmacotherapy

Retrospective studies have investigated weight loss associated with obesity pharmacotherapy in general among patients with a history of bariatric surgery without identifying specific AOM monotherapies or AOM combination therapies. A trend toward greater weight loss has been observed with two or more AOMs compared to patients taking zero or one AOM (29). GLP-1-RAs were found to produce significantly greater weight loss than regimens with a non-GLP-1-RA or intensive lifestyle modification alone (30). Among 37 patients ages 21-30 years, AOM regimens sans metformin were found to be more effective than AOM regimens with metformin (19).

Other studies identified factors associated with greater weight loss in the population of patients with medically managed obesity after bariatric surgery. Greater weight loss results were observed with use of AOMs for patients with a history of laparoscopic adjustable gastric band vs. SG (39) and RYGB vs. SG (20, 39, 40), for patients with pre-operative BMI > 36 vs. those with BMI < 36 (39), and for initiation of AOM at the weight loss plateau rather than waiting until weight regain (17, 19, 20, 40).

## Expert opinion

There are no official guidelines on perioperative weight optimization in patients undergoing bariatric surgery, and there is no consensus on the pharmacologic management of weight regain, inadequate weight loss, or weight loss plateau after bariatric surgery. Whether initiation of AOM preoperatively has long-term benefits to mitigate these post-operative weight issues is unknown. When initiated post-operatively, the choice of which AOM to use for weight optimization is an important component in the long-term care of patients with obesity.

There is a paucity of data available to guide pharmacotherapeutic weight management decisions before bariatric surgery and whether this impacts the sustainability of clinically significant weight loss post-operatively. Weight loss in the pre-operative period has proven benefits, including a reduction in the likelihood of post-operative complications after RYGB (41, 42) but not SG (43). However, there was no effect seen in long-term weight loss outcomes. Notably, these studies did not include patients with pre-surgical weight loss using AOMs. Only one study, conducted in patients with BMI > 50, investigated this question of pre-operative weight optimization and demonstrated significantly greater post-operative weight loss at 24 months with pre-operative phentermine/topiramate 7.5-46mg daily (16). Although post-operative weight loss outcomes associated with using other AOMs before bariatric surgery have not been assessed, other AOMs should still be considered if phentermine/topiramate is contraindicated or if an obesity-associated co-morbidity can be concomitantly addressed (e.g., GLP-1-RA in a patient with obesity and diabetes). It is reasonable for providers to prescribe AOMs in the pre-operative period given the potential to improve long-term weight loss outcomes following bariatric surgery.

Weight regain after bariatric surgery most often begins 1-2 years post-operatively (3). Patients should monitor their weight changes and follow up with their weight management providers at regular intervals after bariatric surgery, as the timing of weight regain or WLP will vary. When a patient reaches a WLP, providers should assess for any factors responsible for preventing additional weight loss, such as dietary changes, initiation of an obesogenic medication, or a post-operative anatomic etiology (e.g., gastro-gastric fistula). The period when patients reach a weight loss plateau represents an opportunity to intervene, with or without an AOM, to address obesogenic factors. Several studies have suggested that greater weight loss was achieved when AOMs were initiated at the weight loss plateau rather than waiting for weight regain (17, 19, 40), with one study demonstrating statistical significance (20). If no clear explanation for the WLP is discovered that can be managed by other means (e.g., endoscopic or surgical revision of a gastro-gastric fistula), AOM initiation should be considered at the time of WLP to promote further weight loss and prevent weight regain.

Of all AOMs, liraglutide, topiramate, and phentermine/topiramate are the pharmacologic agents best supported with current observational studies. Use of GLP-1-RAs for weight optimization after bariatric surgery will likely gain popularity given evidence in the post-surgical population (30) and the proven weight loss efficacy of newer agents in the non-surgical population with obesity. Semaglutide and tirzepatide are the newest additions to the family of GLP-1-RAs, with the latter being a dual GLP-1 and GIP receptor agonist. However, there are no publications to date describing the efficacy of semaglutide or tirzepatide in patients who have undergone bariatric surgery. Pharmacotherapies with gut peptide modulators are emerging as the most effective AOMs (44, 45), with the most recent addition, tirzepatide 15 mg, demonstrating about 20% average weight loss, greater than lap band but less than sleeve gastrectomy and gastric bypass (38). Despite the absence of data detailing the use of these newer agents after bariatric surgery, the existing GLP-1-RA data in this setting and the substantial weight loss seen in non-surgical patients with obesity using semaglutide 2.4mg and tirzepatide suggest that these agents will likely provide more post-operative weight loss than prior GLP-1-RAs.

Medical co-morbidities may influence the choice of on- or off-label AOM used for weight optimization after bariatric surgery. In addition to significant weight loss, semaglutide has proven systemic benefits in the non-surgical population (46–50), whereas tirzepatide trials are still ongoing. Semaglutide would be an optimal AOM for patients with post-surgical weight regain, WLP, or inadequate weight loss who also have a history of a stroke, coronary artery disease, non-alcoholic fatty liver disease, or type 2 diabetes. Tirzepatide is FDA-approved for individuals with type 2 diabetes (51) and may be cardioprotective (52), pending official results of its cardiovascular outcome trial (53). As another example, patients who also experience migraine headaches may benefit from topiramate given its FDA-approval as a migraine prophylaxis.

Contraindications should also inform the choice of AOM used for weight optimization after bariatric surgery. Topiramate or phentermine/topiramate are options in patients who have a contraindication to GLP-1-RAs (e.g., personal or family history of medullary thyroid carcinoma) or who have poorly controlled gastroesophageal reflux disease, which is a common concern after sleeve gastrectomy. For patients who are not candidates for GLP-1-RAs and have a contraindication to topiramate (e.g., nephrolithiasis) and/or phentermine (e.g., uncontrolled hypertension), monotherapy bupropion or bupropion/naltrexone might be considered, though little data exists regarding its efficacy after bariatric surgery.

The use of an AOM may not be appropriate or effective for all patients with weight regain, inadequate weight loss, or WLP following bariatric surgery. If a patient’s unexpected post-operative weight trajectory is due to an anatomic etiology, surgical or endoscopic revision may be indicated. After SG and RYGB, revisional bariatric surgery has become the third most common type of bariatric surgery performed in the Unites States (54), with weight regain as the most common indication for revision (55). Revisional surgeries include conversion to a different bariatric operation (e.g., gastric band to SG), re-sleeve after SG, or prolongation of the biliopancreatic limb after RYGB. Endoscopic options for revision include a transoral outlet reduction (TORe) and revisional endoscopic sleeve gastroplasty (ESG), which result in approximately 10-15% additional weight loss at 1 year after revision (56, 57). A TORe aims to decrease the diameter of a dilated gastro-jejunal anastomosis using an endoscopic suture device. Endoscopic suturing is also used during the revisional ESG to reduce the gastric volume in patients with a dilated gastric pouch after SG.

## Conclusion

In patients with a history of bariatric surgery, the presentations of weight regain, weight loss plateau, or inadequate weight loss are opportunities for weight optimization. Observational studies have consistently demonstrated the effectiveness of AOMs in treating these post-operative weight issues, with liraglutide, topiramate, and phentermine/topiramate having the best supporting evidence among all AOMs. The future of perioperative weight optimization in patients undergoing bariatric surgery is an increasingly empowered field due to the availability of on- and off-label AOMs, evolution of gut peptide modulators as pharmacotherapy, and the development of endoscopic interventions.

## Data availability statement

The original contributions presented in the study are included in the article/supplementary material. Further inquiries can be directed to the corresponding author.

## Author contributions

EL: Created first draft and performed edits according to feedback from senior authors OS: Contributed to second draft, primarily within Expect Opinion and Conclusion sections BT: Provided feedback and guidance on all drafts LA: Provided feedback and guidance on all drafts. All authors contributed to the article and approved the submitted version. 
